# Dermal Delivery of Selected Polyphenols from *Silybum marianum*. Theoretical and Experimental Study

**DOI:** 10.3390/molecules24010061

**Published:** 2018-12-24

**Authors:** Pavel Kosina, Markéta Paloncýová, Alena Rajnochová Svobodová, Bohumil Zálešák, David Biedermann, Jitka Ulrichová, Jitka Vostálová

**Affiliations:** 1 Department of Medical Chemistry and Biochemistry, Faculty of Medicine and Dentistry, Palacký University, Hněvotínská 3, 775 15 Olomouc, Czech Republic; pavel.kosina@upol.cz (P.K.); alena.rajnochova@upol.cz (A.R.S.); jitka.ulrichova@upol.cz (J.U.); 2 Regional Centre of Advanced Technologies and Materials, Department of Physical Chemistry, Faculty of Science, Palacký University, tř. 17 listopadu 12, 771 46 Olomouc, Czech Republic; marketa.paloncyova@upol.cz; 3 KTH Royal Institute of Technology, School of Engineering Sciences in Chemistry, Biotechnology and Health, Department of Theoretical Chemistry & Biology, S-106 91 Stockholm, Sweden; 4 Department of Plastic and Aesthetic Surgery, University Hospital Olomouc, I. P. Pavlova 6, 779 00 Olomouc, Czech Republic; bohumil.zalesak@fnol.cz; 5 Institute of Microbiology, Laboratory of Biotransformation, Academy of Sciences of the Czech Republic, Vídeňská 1083, 142 20 Prague, Czech Republic; biedermann@biomed.cas.cz

**Keywords:** *Silybum marianum*, flavonolignans, flavonoids, experimental and computational hydrophobicity, theoretical lipid membrane models, skin intake

## Abstract

Silymarin is a well-known standardized extract from the seeds of milk thistle (*Silybum marianum* L., Asteraceae) with a pleiotropic effect on human health, including skin anticancer potential. Detailed characterization of flavonolignans properties affecting interactions with human skin was of interest. The partition coefficients log *P_ow_* of main constitutive flavonolignans, taxifolin and their respective dehydro derivatives were determined by a High Performance Liquid Chromatography (HPLC) method and by mathematical (in silico) approaches in *n*-octanol/water and model lipid membranes. These parameters were compared with human skin intake ex vivo. The experimental log *P_ow_* values for individual diastereomers were estimated for the first time. The replacement of *n*-octanol with model lipid membranes in the theoretical lipophilicity estimation improved the prediction strength. During transdermal transport, all the studied compounds permeated the human skin ex vivo; none of them reached the acceptor liquid. Both experimental/theoretical tools allowed the studied polyphenols to be divided into two groups: low (taxifolin, silychristin, silydianin) vs. high (silybin, dehydrosilybin, isosilybin) lipophilicity and skin intake. In silico predictions can be usefully applied for estimating general lipophilicity trends, such as skin penetration or accumulation predictions. However, the theoretical models cannot yet provide the dermal delivery differences of compounds with very similar physico-chemical properties; e.g., between diastereomers.

## 1. Introduction

The primary function of the skin is to form a barrier between the body and the environment that is essential for maintaining body homeostasis. The epidermal barrier prevents excessive trans-epidermal loss of water, electrolytes or heat, but also serves as a barrier to the entry of harmful environmental toxic substances and infectious microorganisms or protects the body against damage caused by physical stimuli such as heat or radiation. The outermost layer of the skin, the *stratum corneum*, although very thin provides the main barrier to the transport of most topically applied compounds. The *stratum corneum* is formed from denucleated, keratin-filed, non-living cells called corneocytes that are anchored in a lipid matrix (major components include ceramides, fatty acids, and cholesterol). With its specific composition and morphology, the *stratum corneum* forms a unique biomembrane that is about a thousand times less permeable to water and compounds than other membranes in the body [1,2]. The cutaneous delivery of substances represents an attractive option for the treatment or prevention of various dermatological conditions with minimal side-effects and avoids metabolic biotransformation that may occur in the liver. However, the effective barrier function of the skin limits the use of many compounds as transdermal agents [2]. Therefore, a promising candidate for dermatological application has to cross the *stratum corneum* barrier and reach viable cells in the deeper layers of the epidermis and dermis.

**Figure 1:**
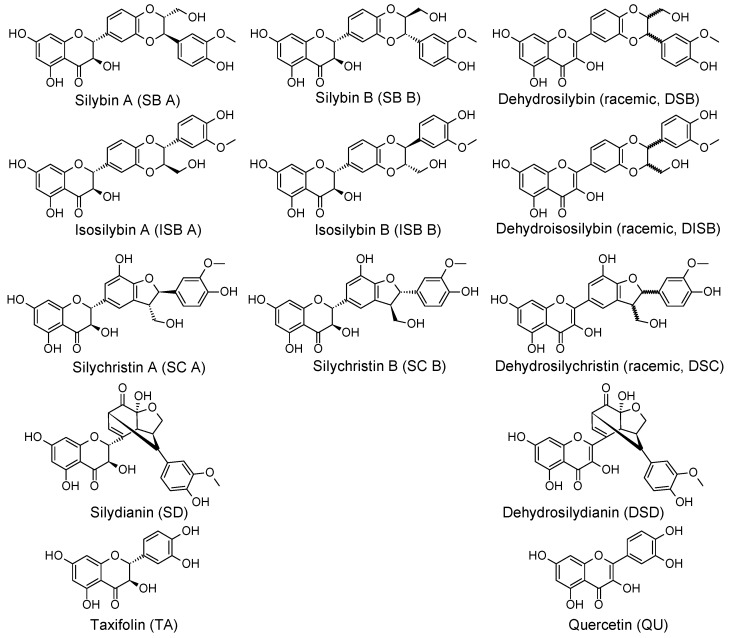
Structure of studied polyphenols.

Silymarin (SM) is a multicomponent extract from the seeds of the milk thistle (*Silybum marianum* L., Asteraceae). *S. marianum* is one of the oldest known herbal plants, and has been widely used in traditional European medicine for over two thousand years, especially for treating liver disorders. The main polyphenolic component of SM is the flavonolignan silybin (SB) [3]. Other components in considerable amounts include isosilybin (ISB), silychristin (SC), silydianin (SD) and their flavonoid precursor taxifolin (TA). Dehydrosilybin (DSB) is also present in small amounts [4]. The studied compounds’ structures, including available diastereomers, are presented in Figure 1. 

Besides the above-mentioned congeners of silybin, a lot of minor flavonolignans have been identified to date [5,6,7]. Multiple biological actions including antioxidant, anti-inflammatory, immunomodulatory or cytoprotective, make SM and SB attractive for dermatological applications. Therefore, the beneficial effects of SM and SB have been studied on skin carcinogenesis induced chemically [8] or by chronic exposure to UVB light [9], on skin disorders such as contact dermatitis [10], skin hyperpigmentation (melasma) [11], and wound healing [12]. Although many in vivo or clinical studies with SM/SB topical application were conducted, there is minimal information on the skin absorption and permeability (bioavailability) of SB and other SM components.

Efforts to characterize the interactions of compounds with biological membranes have led to an investigation of the hydrophobicity of the utilized molecules [13,14]. Apart from experimental methods, a significant amount of work has been done on in silico predictions in recent years. Hydrophobicity in terms of the widely used *n*-octanol/water partition coefficient log *P_ow_* can be predicted by structure-based tools, such as the freely available AlogP [15], Molinspiration [16] etc. Most of these methods use topological descriptors, or the fragmentation of molecules to functional groups or atoms. From fitting a large number of available experimental data, the effects of the molecular fragments present in molecules on their log *P_ow_* can be estimated. These methods are strictly focused on log *P_ow_* and work well, especially among molecules similar to the training set. Quantum-calculation based methods or molecular dynamics simulations are another possibility for in silico predictions. These methods are more general and usually not focused on the prediction of a single property. Their performance in terms of log *P_ow_* can be worse, but they are not limited to a single case or a certain molecular type. Currently available tools for log *P_ow_* prediction have been summarized in a review by Tetko and Poda [17].

The aim of this study was (i) to determine the partition coefficients log *P_ow_* of selected polyphenols of SM (SB, ISB, SD, DSB, SC, TA) as well as dehydroderivatives dehydrosilychristin (DSC), dehydrosilydianin (DSD), dehydroisosilybin (DISB), and quercetin (QU) were included in the study (see Figure 1) by High Performance Liquid Chromatography (HPLC) and by mathematical (in silico) approaches in an *n*-octanol/water system and to compare the obtained values; (ii) to calculate the permeability coefficients of selected SM components for model membranes (bilayer of dioleoylphosphatidylcholine, ceramide or mixture ceramide, cholesterol and lignoceric acid) and to compare them with experimental data obtained using human skin fixed in Franz diffusion cells. 

## 2. Results and Discussions

### 2.1. Experimental Partition Coefficients log P_ow,exp_

The partition coefficients (log *P_ow,exp_*) of studied polyphenols including available diastereomers were obtained using reverse phase HPLC in the pH range of 4.5–8.5. For evaluating the studied compounds’ hydrophobicity, log *P_ow,exp_* of all reference substances were used except for aniline, whose log *P_ow,exp_* value (0.9) was conspicuously lower than that of the other reference substances (their individual log *P_ow_* and applied amounts are presented in Appendix Table A1). Aniline was only used to evaluate the log *P_ow,exp_* of TA at pH 8.5 due to a big negative shift in the TA log *P_ow,exp_* value. 

The relation between the capacity factor *k* and log *P_ow_* of reference compounds at various pH of mobile phase, employing quadratic equations, was evaluated. The recommended application of the linear equation [14] based on reference compound capacity factors (retention times) and log *P_ow_* values for evaluating the test compounds’ log *P_ow_* gave only an r^2^ value of 0.89, see Table A2. The application of the quadratic equation resulted in obtaining more valuable data for the studied compounds’ log *P_ow,exp_* evaluation (r^2^ ~ 0.99), see Table A2. Therefore, the quadratic equations were employed to estimate the log *P_ow,exp_* values of the studied compounds. 

A minimal influence of temperature (25 °C ~ recommended by OECD107 [13]) vs. 32 °C ~ corresponding to the skin surface temperature) on log *P_ow,exp_* values was found (data not shown). Similarly, a minor effect of temperature was found for theoretical (calculation) models (data not shown). The log *P_ow,exp_* values of the studied polyphenols, including data for diastereomers, were evaluated in 5 mM phosphate (Table 1), additionally in 5 mM formate and 5 mM acetate buffer as well, in a gradient with methanol at 25 °C with the pH range of 4.5–8.5. A minimal effect of mobile phase composition (phosphate vs. formate vs. acetate) was found (Table 1, Table A3 and Table A4). Application of acetate or formate buffers instead of phosphate allows mass spectrometry identification of studied compounds.

Of all the studied compounds, the lowest log *P_ow,exp_* values were found for TA and the highest ones for DISB at all pH values. Among flavonolignans and their dehydro derivatives, SC A exhibited the lowest log *P_ow,exp_* value. The hydrophobicity of flavonolignans in increasing order was as follows: SC A < SD < SC B < DSD < SB A < SB B < DSC < ISB A < ISB B < DSB < DISB. Flavonolignan dehydro derivatives were more hydrophobic than the corresponding parent substances. The same effect was observed with the pair of flavonoids TA and QU. The increasing pH value of the mobile phase decreased the log *P_ow,exp_* value for all polyphenols (Table 1). 

### 2.2. Calculated Partition Coefficients log P_ow,calc_

The computational tools for lipophilicity evaluation (log *P_ow,calc_*), AlogP [15], Molinspiration [16] and COSMOtherm 15 [18] showed a slightly different order of the molecular hydrophobicity of the studied compounds compared to the experimental evaluation, but the general trends remained the same (Table 2). 

**Figure A1:**
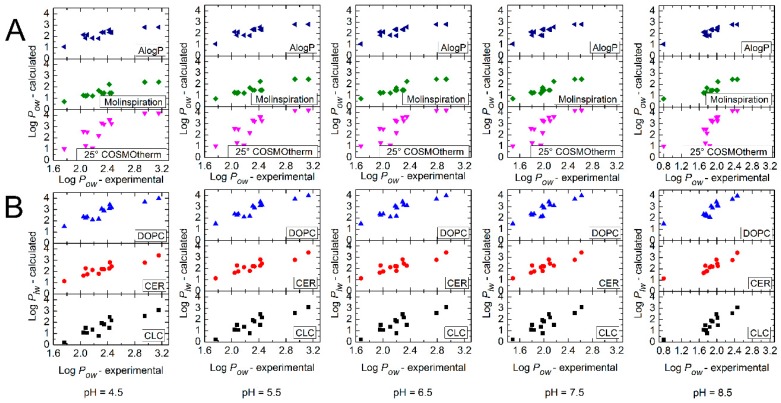
Relation between (**A**) calculated log *P_ow,calc_* and experimental log *P_ow,exp_* measured in phosphate buffer at pH 4.5–8.5; (**B**) calculated log *P_lw,calc_* values and experimental log *P_ow,exp_* measured in phosphate buffer at pH 4.5–8.5.

TA was the least lipophilic molecule and DISB was the most hydrophobic. Also, the dehydro derivatives (DISB, DSB, DSC and QU) were more lipophilic than their respective parent substances, which agrees with experimental data. The AlogP tool predicted log *P_ow,calc_* values for the studied compounds within the range of 1.1–2.8 and the Molinspiration tool computed log *P_ow,calc_* values within the range of 0.7–2.4. Using COSMOtherm 15, a wider range of log *P_ow,calc_* values were obtained (1.0–4.2) than with the above-mentioned tools (Table 2). The correlation of the experimental (log *P_ow,exp_*) and calculated (log *P_ow,calc_*) data in terms of linear fit, correlation coefficient (r^2^) and mean differences (MD) was evaluated (for data see Appendix Figure A1 and Table A5).

The lowest r^2^ values were found with COSMOtherm 15 and the highest with Molinspiration (pH 4.5–7.5) and AlogP (pH 8.5). The lowest MD were obtained with AlogP over the whole pH range (Table A5). 

### 2.3. Lipid/Water Partition Coefficients log P_lw,calc_

Apart from *n*-octanol/water partition (log *P_ow,calc_*), lipid/water partition coefficients (log *P_lw,calc_*) in membrane models (dioleoylphosphatidylcholine (DOPC), ceramide (CER) and ceramide, lignoceric acid and cholesterol mixture (CLC)) were calculated using COSMOmic (Table 2). The best correlation between log *P_lw,calc_* and log *P_ow,exp_* was obtained using the DOPC membrane model, where r^2^ reached up to 0.84 with respect to the data at pH 4.5 and 5.5. The correlation of log *P_lw,calc_* values for CER and CLC membrane models with experimental data was lower than for the DOPC membrane model, as the r^2^ values varied from 0.75 to 0.82. In terms of MD, the CER membrane model performed best (MD in the range of 0.24 to 0.37 over the whole pH range). In the DOPC membrane model, the MD increased with pH, while in the CLC membrane model the MD decreased with pH. Although working on a very different principle, the CER membrane model appeared to work with a similar level of accuracy to AlogP in terms of MD and together with the DOPC membrane model scored very well in terms of r^2^ (for data see Appendix Figure A1 and Table A5).

### 2.4. Skin Permeability of Polyphenols

The skin permeability of polyphenols was studied using Franz diffusion cells with human skin. None of the studied compounds were found in the acceptor liquid, however all were found in the tissue. The skin intake of the studied compounds during transdermal transport was influenced by the ethanol content and pH of the donor liquid. The results are presented in Table 3 and expressed as the amount of individual polyphenols in pmol per g of skin tissue per 24 h.

Apart from DSB, the skin content of all polyphenols was lower when applied in donor liquid with pH 8.5 than pH 6.5. When donor liquid with pH 6.5 was used, the skin intake of polyphenols was in the order: TA < SD < SC < DSB < SB < QU < ISB and at pH 8.5 it was TA < SD < SC < QU < SB < ISB < DSB. For the main silymarin constituent (SB), both diastereomers SB A and SB B were determined. The amount of SB B in skin was found to be slightly higher than that of SB A (data not shown). The skin content of QU dramatically decreased (~8.5 times), when QU was applied in donor liquid with pH 8.5. 

**Figure 2:**
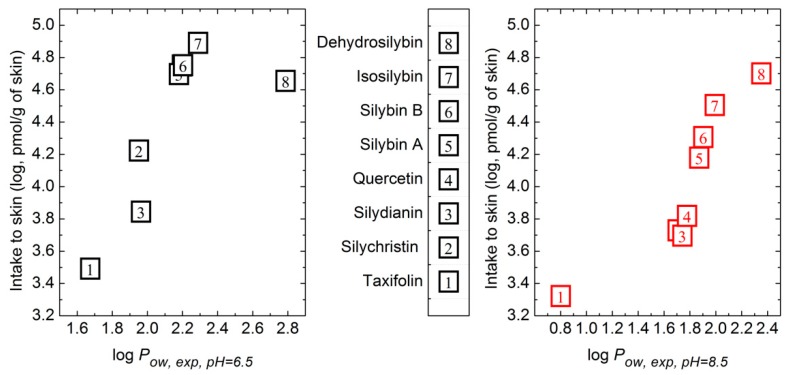
Relation of compounds’ skin intake to their lipophilicity. The relation of skin intake to experimental *n*-octanol/water partition coefficient (log *P_ow,exp_*) at pH 6.5 is presented on the left; the relation of skin intake to log *P_ow,exp_* at pH 8.5 is shown on the right.

Increasing ethanol content had a mostly negative effect on the polyphenol penetration into the skin (Table 3). The skin bioavailability of a substance is usually predicted by its log *P_ow_* value [2]. Therefore the log *P_ow,exp_* values were compared with the skin intake of polyphenols. At pH 6.5, the correlation coefficient r^2^ of log *P_ow,exp_* and logarithm of skin intake varied between 0.54 to 0.60 (see Appendix B—Table A6). However, inspecting the data (Figure 2), DSB was identified as an outlier; after its exclusion the r^2^ between log *P_ow,exp,_* at pH 6.5 and the logarithm of the measured skin intake reached 0.94 (Table A6). The log *P_ow,exp_* values at pH 8.5 were not as suitable for estimating the skin intake as the data obtained at pH 6.5. Generally, the highest correlation was observed for ethanol-free systems.

### 2.5. Calculated Permeability of Polyphenols log Perm

**Figure 3:**
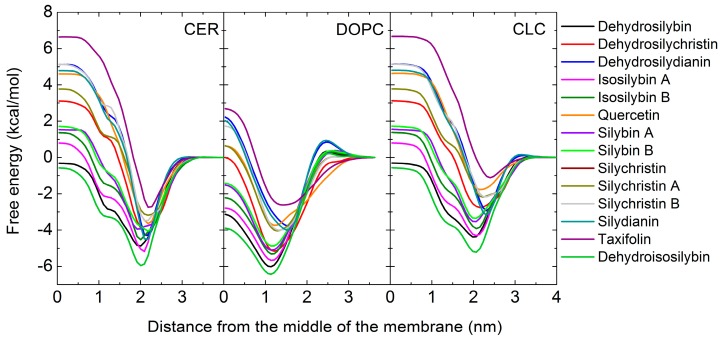
Free energy profiles of studied compound in ceramide (CER), dioleoylphosphatidylcholine (DOPC) and ceramide, lignoceric acid and cholesterol mixture (CLC) membrane models.

From the free energy profiles on all membrane models (CER, CLC and DOPC, see Figure 3) obtained by COSMOmic, the permeability (log *Perm*) of polyphenols were calculated (Table 4). The order of free energy profiles of studied polyphenols (Figure 3) as well as a penetration barrier (Δ*G^pen^)* and *Perm* (Table 4) corresponds to lipophilicity (log *P_lw,calc_*), calculated by theoretical tool (COSMOmic), see Table 2. The lowest free energy profiles were found for DISB and DSB; and the highest one for TA. 

### 2.6. Calculated Partition Coefficient log P_lw,calc_ and Permeability log Perm Correlation with Polyphenols’ Skin Intake

**Figure 4:**
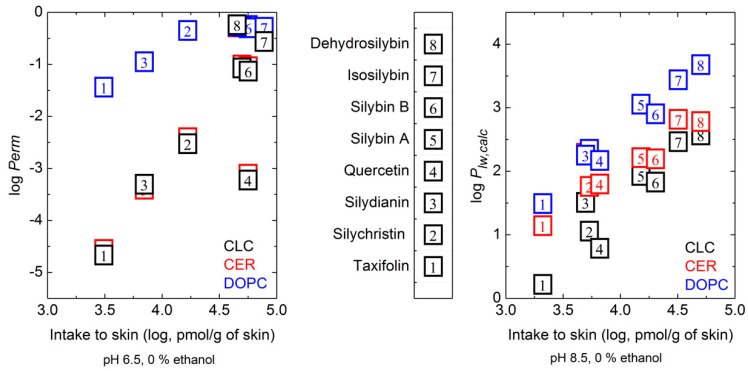
Relation between compounds’ skin intake and calculated permeability or partition coefficients in lipid membrane models. The relation between the logarithms of the compounds’ skin intake at pH 6.5 and calculated permeability (log *Perm*) in lipid membrane models is presented on the left; the logarithms of the compounds’ skin intake at pH 8.5 and calculated lipid membrane models partition coefficients (log *P_lw,calc_*) is shown on the right. CER—ceramide, DOPC—dioleoylphosphatidylcholine, CLC—ceramide, lignoceric acid and cholesterol mixture.

The experimental skin intake was compared to log *P_lw,calc_* and log *Perm* (for data see Appendix B—Table A7). For the skin intake at pH 6.5 (no ethanol) after excluding QU, a better correlation was found with log *Perm* in CER and CLC membrane models (r^2^ = 0.95 and 0.94). The DOPC membrane model did not distinguish well between more hydrophobic compounds, the correlation was lower. For the skin intake at pH 8.5, a correlation was found with log *P_lw,calc_* for all the membrane models used (Figure 4). 

Of the computative log *P_ow_* tools used, the best prediction of log *P_lw,calc,_* was obtained with the DOPC membrane model (r^2^ = 0.97). The ethanol content and/or pH increase in the donor liquid influenced the correlation between the skin intake (experimental data) and log *Perm* or log *P_lw,calc_*. For both pH values and ethanol content (5 and 10% (*v*/*v*)) a decrease in r^2^ was observed. At pH 6.5 and an ethanol concentration of 15% (*v*/*v*) an increase in r^2^ was found for all membrane models (for data see Appendix B Table A7). A clear linear dependence was found between log *Perm* and the skin intake for the less hydrophobic compounds (TA, SD, SC). The calculation was unable to clearly distinguish between the highly hydrophobic compounds, as *Perm* depended exponentially on the highest free energy. The hydrophobic compounds with high log *P_lw,calc_* (affected primarily by the lowest free energy well—see Material and Methods section: Design of computational of hydrophobicity of polyphenols)—did not show any significant dependence of the skin intake on log *Perm* at the DOPC membrane. For other membrane models, where the penetration free energy barrier is higher than with DOPC (Figure 3, Table 4), a dependence of skin intake on log *Perm* over the whole range was found, however with two outliers, DSB and QU. 

### 2.7. Discussions

Despite the use of silymarin in dermatological preparations, a limited amount of information about the hydrophobicity of silymarin’s polyphenols is available. Our study focused on characterizing the hydrophobicity of selected flavonolignans (including diastereomers and dehydro derivatives) and the flavonoids taxifolin and quercetin and their interaction with model membranes and human skin using theoretical and experimental tools. 

For compounds with a possible dermal application, it is essential to be able to cross the lipids layer barrier of the *stratum corneum* and be soluble in both hydrophobic and hydrophilic environments. These ambivalent properties can be evaluated by the *n*-octanol/water partition coefficient [2]. The official OECD method, based on the partition of a studied compound between the water and *n*-octanol phases (*n*-octanol/water partition coefficient; log *P_ow_*) measured by the shake flask method, imitates the compound’s interaction with the biomembrane very well [13]. In this way the log *P_ow_* also characterizes the partitioning of a compound between the lipophilic *stratum corneum* and the underlying hydrophilic living cells of the epidermis [2]. Today, instead of the shake flask method, another OECD Guideline, Test No. 117 using isocratic HPLC elution was applied [14], but in gradient elution mode [19]. The methods for log *P_ow_* evaluation are based on a comparison of the log *P_ow_* values of a set of reference compounds and their retention times (capacity factors) in a C_18_ chromatographic column with retention times and capacity factors of studied compounds [14]. The application of the HPLC method for estimating the log *P_ow_* values of polyphenols has several advantages: a stable temperature during analysis, the application of small amounts of pure studied compounds as well as their mixtures with similar structure and/or similar physico-chemical properties. By the HPLC gradient elution method, the mixture of main constitutive *S. marianum* flavonolignans (SB, SC, SD, ISB), including diastereomers for SB, SC and ISB, and their respective dehydro derivatives (eleven individual compounds) as well as TA and its dehydro derivative QU could be separated in one run and the obtained data were suitable for the evaluation of log *P_ow,exp_* values (Table 1). 

Data for individual diastereomers of SB, SC and ISB as well as for DISB are published here for the first time. The log *P_ow,calc_* values that we found for QU and TA (Table 2) are in agreement with reports by Maroziene et al. [20]. The log *P_ow,exp_* value of QU corresponds to the data by Rothwell et al. [21]. Similarly, the log *P_ow,exp_* values for the pair of flavonolignans DSB and SB obtained by the shake flask method published by Gažák et al. [22] agree with our data obtained by the HPLC method. Here we further found that the hydrophobicity of SM’s compounds decreased with increasing pH in all applied solutions (mobile phases), which corresponds to the results for TA, SB, ISB, SC, SD obtained by the shake flask method [23]. A small effect of pH on log *P_ow,exp_* was observed at pH from 4.5 to 6.5. An obvious decrease in hydrophobicity (log *P_ow,exp_*) was found from pH 6.5 to 8.5 (Table 1). This phenomenon is connected with the ionization of ionizable groups in the studied compounds. The pK_a1_ value of SB, SC, SD [24], TA [25] and QU [26] is close to neutral pH (~7.0) and pK_a2_ is ~8.5. The strongest decrease in log *P*_ow_ in the case of TA at pH 8.5 relates to its pK_a_ values (pK_a1_ 7.1; pK_a2_ 8.6; pK_a3_ 8.59; pK_a4_ 11.82) [25] as well. Generally, the neutral form of the molecule is more lipophilic than the deprotonated one. The order of the log *P_ow,exp_* of the studied polyphenols (TA, SB, SC, SD and ISB) was not the same as that found by Zeng et al. [23]. It could be caused by the experimental limitations of the shake flask method. Studied compounds can occur in both phases and also in an interphase; this phenomenon is eliminated in the reverse phase chromatography method used in our study. 

The experimental evaluation of compound hydrophobicity is time-consuming, experimentally (the shake flask method) and financially (reverse phase HPLC based approaches) demanding and also unfavorable to the environment (does not follow green chemistry approaches) [19]. Currently a large number of sophisticated computational tools, based on various parameters, are available [17,27,28]. These in silico methods are helpful for screening a large set of compounds and for basic behavior classification in terms of compound hydrophobicity and bioavailability, reducing the amount of in vivo experiments or clinical trials [29]. On the other hand, the theoretical methods have some limitations, including the inability to properly simulate all situations in a real biological system (i.e., skin). In our study the tools COSMOtherm 15, AlogP and Molinspiration were used. COSMOtherm 15 is generally focused on the partitioning between fluid phases and not directly on log *P_ow_*, and does not consider the pH effect; therefore, it scored worst of the calculation methods used. At pH 4.5–7.5, the highest correlation was obtained with Molinspiration and at pH 8.5 with AlogP. This may be caused by AlogP’s use of electrotopological state descriptors [30]. This is an atomic approach that also considers the valence state of the atoms, which may better reflect the experimental molecule ionization at pH 8.5 (corresponding to the pK_a_ of studied polyphenols). AlogP and Molinspiration tools are based on a large set of experimental log *P_ow_* values and both are applicable around neutral pH. None of the theoretical tools used were able to reflect the behavior of the set of polyphenols over the whole pH range (4.5–8.5). According to the correlations obtained, the choice of optimal log *P_ow_* calculation tool should consider the desired real experimental conditions ~ the real applicative conditions of the compound. When the studied polyphenols are in ionized form (pH over pK_a_) only the AlogP tool is able to estimate log *P_ow,calc_* values. At pH below pK_a_, Molinspiration described the system best. The replacement of *n*-octanol with lipid membrane models (DOPC, CER or CLC) in COSMOmic improved the prediction strength. COSMOtherm and COSMOmic inputs are a 3D structure of the compound (conformational variability is considered) [18] and thus differentiation between diastereomers A and B is possible. The differences in the hydrophobicity of the diastereomers were too small to distinguish them, and this ability remains a benefit of experimental evaluation. ALogP and Molinspiration tools do not work with diastereomerism. 

Although *n*-octanol/water partitioning log *P_ow_* is taken as a gold standard for lipophilicity estimation [19], considering the lipophilicity directly in terms of the lipid/water partition coefficient log *P_lw_* predicts it better. However, using theoretical approaches to characterize the transdermal delivery in vivo is still complicated due to the complexity of the systems in terms of composition or the real interaction between human skin tissue and a topically applied compound. Therefore, both approaches were applied in our study. Many papers have demonstrated that increasing lipophilicity increases the skin permeation of compounds, and a log *P_ow_* of 2–3 seems to be optimal. It is likely that these molecules with intermediate lipophilicity can permeate via both the lipid and polar microenvironments in the intercellular route [31]. Accordingly, most of the studied polyphenols are good candidates for dermal application, especially at acidic pH (4.5–6.5), see Table 1. Our transdermal transport data demonstrated that none of the studied compounds were able to penetrate through the skin, as none of them were detected in the acceptor liquid. However, all the polyphenols were found in the human skin (Table 3). In this way the human skin works as a trap and only the skin intake (accumulation) of polyphenols can be measured. QU skin intake should be taken with care, as the presented results on human skin were affected by QU instability in aqueous solutions that was previously reported [32]. Thus, with QU it is not possible to ensure a constant QU concentration in the donor liquid during experiments (24 h) as with the other studied polyphenols. Due to QU decomposition, the amount in the skin is most likely significantly reduced. A higher instability of QU occurs in alkaline pH [32], which corresponds with a lower amount of QU in the skin at the higher pH of 8.5 in our experiments (Table 3). Therefore, correlations of the polyphenol skin intake with log *Perm* and log *P_lw,calc_* were performed without QU. 

Although the long-term practical use of silymarin, flavonolignan skin delivery is poorly documented. The previous work [33] that studied the in vitro delivery of SB, SD and SC using native, chemically, and physically modified mouse skin does not agree with our results (Table 3). In the mouse models, the flavonolignans were able to penetrate through the skin into the acceptor liquid (SB > SD > SC). However, the human skin has a higher barrier function (more robust *stratum corneum* and thicker epidermis) than mouse skin and so mouse skin is not a fully accepted model to mimic the penetration of compounds through the human skin [34]. Nevertheless, SB deposition in mouse skin was notably higher than those of SC and SD [33], which corresponds to our data (Table 3). The order of polyphenol permeation through CER and CLC membrane models (Table 4) also partially agrees with the published data on mouse skin [33]. According to our data on the experimental skin uptake, the studied polyphenols can be divided into two groups: low intake (TA, SC, SD) and high intake (SB, DSB, ISB). This division also corresponded to their hydrophobicity based on theoretical data (log *P_ow,calc_*, log *P_lw,calc_*) and experimental log *P_ow_* values as well. Our results further showed a significant effect of pH on the skin intake of flavonolignans and flavonoids. All these properties may be important for designing dermal preparations containing these polyphenols.

## 3. Materials and Methods

### 3.1. Studied Compounds

Pure flavonolignans (SB, SC, SD and ISB) and their respective oxidized products (dehydrosilychristin (DSC), dehydrosilydianin (DSD), dehydroisosilybin (DISB) and dehydrosilybin (DSB)) were prepared at the Institute of Microbiology, Academy of Sciences of the Czech Republic, Prague, Czech Republic according to previously published studies. For SB and ISB, individual diastereomers were available [35]. SC (natural mixture of diastereomers) and SD were isolated from silymarin by Sephadex LH-20 column chromatography (Sigma Aldrich, Prague, Czech Republic) as described previously [36]. DSB was prepared as described by Gažák et al. [37]. The preparation of other dehydro derivatives was published elsewhere [38]. Flavonoids TA and quercetin (QU) were purchased from Sigma Aldrich (Prague, Czech Republic).

#### Studied Compound Solutions for Hydrophobicity Evaluation

The stock solutions of the studied compounds (1 g L^−1^) were prepared in methanol. To minimize the coelution of analytes during HPLC separation, two mixtures of the studied compounds, at the final concentration of 10 mg L^−1^, were prepared. Mixture 1 contained SD, diastereomer A of silybin (SB A), diastereomer A of isosilybin (ISB A), DSC, DSD and DISB. Mixture 2 contained TA, mixture of diastereomers A and B of silychristin (SC A and B), QU, diastereomer B of silybin (SB B), diastereomer B of isosilybin (ISB B) and DSB. 

### 3.2. Chemicals

Aniline, phenol, 4-chloroaniline, nitrobenzene, benzene, trichloroethylene, toluene, chlorobenzene, and naphthalene, all p.a. grade, were obtained from Lach-Ner (Neratovice, Czech Republic). Buffer components, Dulbecco’s Modified Eagle medium without phenol red, and other chemicals were obtained from Sigma-Aldrich (Prague, Czech Republic). Methanol and acetonitrile, both HPLC gradient grades, were obtained from Merck (Darmstadt, Germany). All solutions were prepared using reverse-osmosis deionized water (Ultrapur, Watrex, Prague, Czech Republic). Nitrogen (99.999%) was obtained from SIAD Czech (Prague, Czech Republic) and helium (99.999%) from Linde Gas (Prague, Czech Republic). 

#### Reference Compounds for Hydrophobicity Evaluation

Reference compounds (aniline, phenol, 4-chloroaniline, nitrobenzene, benzene, trichloroethylene, toluene, chlorobenzene, and naphthalene) for partition coefficient (log *P_ow_*) evaluation by chromatographic method were dissolved in methanol and diluted to the appropriate concentration (Table A1). Finally, the mixture of reference compounds was injected into the chromatographic column.

### 3.3. HPLC System for Hydrophobicity Evaluation

The HPLC chromatographic system Dionex UltiMate 3000 (Dionex Corp., Sunnyvale, CA, USA) consisting of a degasser (SRD-3400, 4 DEGASSER CH), a binary pump (HPG-3400SD), an autosampler (WPS-3000 TSL ANALYTICAL), a column compartment (TCC-3000RS) and a diode array detector (DAD-3000, 190–400 nm) was equipped with a Purospher Star RP-18e, 55 × 2 mm, 3 µm chromatographic column (Merck, Darmstadt, Germany). The log *P_ow_* values of reference compounds, flavonolignans, TA and QU were determined in gradient elution with mobile phase A: 5 mM phosphate (or 5 mM formate or 5 mM acetate buffer, respectively) with 5% MeOH (*v*/*v*); in gradient with the mobile phase B: 100% (*v*/*v*) MeOH (linear gradient elution (% B, *v*/*v*): 0 min (0% B), 22 min (65% B), 25 min (65% (*v*/*v*) B), 25.1 min (0% (*v*/*v*) B), 30 min (0% (*v*/*v*) B). The pH of mobile phase A was set to 4.5, 5.5, 6.5, 7.5, and 8.5 for each utilized buffer. The flow rate was 0.3 mL min^−1^, the injection volume was 10 µL. The temperature of the autosampler as well as the column oven was set to 25 or 32 °C. Detection was carried out at 254 nm. The dead and retention time of the reference compounds as well as studied compounds were measured. The analysis was carried out at least twice.

### 3.4. Hydrophobicity Evaluation

The hydrophobicity (lipophilicity) of compounds was evaluated as the partition coefficient (*P_ow_*), defined as the ratio of the equilibrium concentrations of the dissolved substance in *n*-octanol and water [14].

(1) $$\log P_{ow}=\log\;\frac{C\;n–octanol}{C\;water}$$

The gradient reverse phase HPLC method [19] using a C_18_ column was used to estimate the log *P_ow_* of each studied compound by comparing its retention time (*t_r_*) with the *t_r_* of reference substances with known log *P_ow_*. The capacity factor (*k*) for the reference compound is calculated using the *t*_r_ of reference compound and the dead time of analysis (*t*_0_):

(2) $$k=\frac{t_{R}-t_{0}}{t_{0}}$$

A relation between *k* and log *P_ow_* is usually shown in the following equation, which is used for evaluating the log *P_ow_* of a studied compound [19]:

(3) $$\log P_{ow}=a+b\times \log\;k$$

However, for a more accurate relation between *k* and log *P_ow_*, a polynomial equation of degree 2 (quadratic equation) was applied.

(4) $$k=a\times {\left(\log P_{ow}\right)}^{2}+b\times \log\;P_{ow}+c$$

For evaluating the effect of pH on *P_ow_* value, mobile phases A with various pH levels (4.5; 5.5; 6.5; 7.5, and 8.5) were utilized. Two temperatures (25 and 32 °C) were used during the HPLC analysis.

### 3.5. In Vitro Skin Penetration (Transdermal Transport)

Breast tissue specimens were obtained from healthy women undergoing plastic surgery at the Department of Plastic and Aesthetic Surgery (University Hospital in Olomouc). The use of skin tissue complied with the Ethics Committee of the University Hospital in Olomouc and Faculty of Medicine and Dentistry, Palacký University, Olomouc (date: 6.4.2009, ref. number: 41/09). All patients had given their written informed consent. The skin fragments were transported in phosphate buffered saline (PBS) containing antibiotics (penicillin (500 mg mL^−1^), streptomycin (500 U mL^−1^) and amphotericin B (1.25 mg mL^−1^)). The skin was then washed with PBS three times and used as a membrane in Franz cells. The *stratum corneum* was oriented into the donor compartment. Liquid Dulbecco’s Modified Eagle medium without phenol red and antibiotics was used as the acceptor. The donor compartment was filled with the studied compounds (2 mL; 50 μM) in phosphate buffer (50 mM, pH = 6.5 or 8.5) with various concentrations of ethanol (0, 5, 10 or 15% (*v*/*v*)). The available diffusion area between the donor and acceptor compartment was 1.77 cm^2^. The donor compartment and sampling port was closed with parafilm to protect those compartments against evaporation during the experiment. The Franz cells were incubated in a water bath to guarantee a temperature of the donor liquid of 32 °C (temperature of on the human skin surface) and the stirring rate of acceptor liquid was 200 rpm. To minimize photodecomposition of test compounds, the incubation was carried out in the dark. 

After 24 h, the aliquots of donor and acceptor liquids were collected. The skin was removed from the Franz cell and washed with PBS and cleaned and dried with a cotton mull. The skin tissue was weighed, cut with scissors into small pieces, mixed with an extraction mixture (ACN/MeOH/H_2_O, 50:40:10, *v*/*v*/*v*) in the ratio 1:3, and homogenized for 3 min at 21,500 rpm using a ULTRA-TURRAX^®^ T 25 basic IKA^®^ with a S 25 N-10 G dispersing element (IKA^®^-Werke Staufen, Germany). The homogenate was centrifuged (12,000× *g*, 10 min, 4 °C). The supernatant was then diluted with HPLC mobile phase A in the ratio 1:1 (an 8-fold final dilution). The donor liquid was finally diluted with HPLC mobile phase A in the ratio 1:9. The acceptor liquid was diluted with HPLC mobile phase A in the ratio 1:1. After centrifugation (12,000× *g*, 10 min, 4 °C), samples were analyzed by HPLC (see below).

### 3.6. HPLC/HESI-MS of Selected Flavonolignans, Taxifolin and Quercetin in Biological Matrices

The HPLC chromatographic system was the same as for the hydrophobicity study (Section 3.3) with the following modifications: the monolithic chromatographic column Chromolith Performance RP-18e, 100 × 2 mm and monolithic guard column RP-18e, 5 mm × 2 mm (Merck, Darmstadt, Germany) were utilized for the analysis of individual selected flavonolignans/flavonoids in gradient elution with mobile phase A: MeOH:H_2_O:CH_3_COOH (37:63:0.5); in a gradient with mobile phase B: 100% MeOH (linear gradient elution (%, *v*): 0–1 min (0% B), 1–3 min (100% B), 3–5 min (100% B), 5–5.1 min (0% B), 5.1–8.5 min (0% B)). The flow rate was 0.3 mL min^−1^, the injection volume was 10 µL. Before HPLC/HESI-MS analysis, all samples were kept in dark. The temperature of the autosampler was set to 10 °C, and the column oven was set to 30 °C. The HPLC system was on-line connected to the quadrupole ion-trap MS instrument LCQ Fleet (Thermo Scientific, Waltham, MA, USA) operating in a negative as well as a positive heated electron spray ionization (HESI) mode. The HESI-MS parameters selected were: heater temperature 100 °C, capillary temperature 295 °C; in negative/positive mode: spray voltage 2.9/4.0 kV, and capillary voltage −20/+31 V. Nitrogen was used as the sheath, auxiliary and sweep gas, and helium was used as the collision gas. The sheath, auxiliary and sweep gas flow rates were 35, 5, and 1 (in arbitrary units). The MS spectrum was monitored in the range 150–1000 *m*/*z* for both ionization modes. The MS^2^ fragments were isolated from the following parent ions for the negative mode: 301, 303, 479, and 481 *m*/*z*, respectively; for the positive mode: 303, 305, 481, and 483 *m*/*z*, respectively. The normalized collision energy was 25. The quantification of selected flavonolignans, TA, and QU was carried out according to a calibration curve in the range of 0.1–100 ng per injection.

### 3.7. Design of Computational Evaluation of Hydrophobicity of Polyphenols

**Figure 5:**
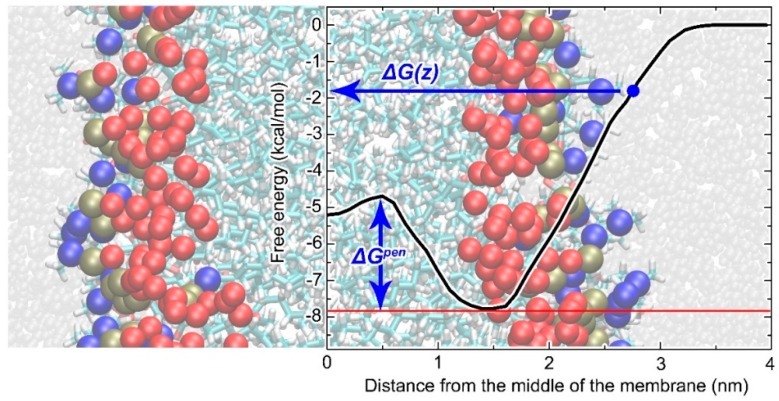
Structure of model membrane and free energy profile of penetration barrier. Lipid membrane constituents: the hydrocarbon chains in the membrane core are represented as cyan and white sticks; oxygen, phosphorus and nitrogen atoms in the DOPC membrane are shown as red, olive and blue balls; water molecules are represented as grey balls. The graph shows the free energy profile of the penetration barrier (Δ*G^pen^*) and the free energy at depth *z* Δ*G*(*z*).

In order to obtain a computational prediction of the hydrophobicity of flavonolignans, TA, and QU, the *n*-octanol-water partition coefficients were calculated (log *P_ow,calc_*) with the commercial software COSMOtherm 15 [18] based on quantum-chemical calculations and by the freely available online tools AlogP [15] and Molinspiration [16]. AlogP and Molinspiration are based on the analysis of available experimental data; specific conditions such as temperature, pH, etc. cannot be chosen. Data can be interpreted as an estimation of log *P_ow,calc_* for mean conditions in which the original thousands of data were obtained. Details about the computational methods used can be found; e.g., in the review by Tetko and Poda [17]. In contrast, in COSMOtherm 15, the parameters for the calculation can be set up. Log *P_ow,calc_* as a partition coefficient between two phases—water (unionized form) and wet *n*-octanol (*n*-octanol saturated with water; 27.4% of mass is formed by water, rest is *n*-octanol)—were calculated at 25 and 32 °C. More details about the calculations can be found in Appendix C. The COSMOmic [39] tool was used for lipid/water partition coefficient calculation (log *P_lw,calc_*). Free energy profiles along the axis perpendicular to the membrane plane from water to the middle of the membrane (Figure 5) were calculated. This can be used further for calculations of log *P_lw,calc_* or permeability (*Perm*):

(5) $$P_{lw,calc}=\overset{n}{\underset{0}{\int }}\left(e^{-\frac{\Delta G\left(z\right)}{RT}}-\frac{\rho_{\left(z\right)}^{water}}{\rho_{\left(n\right)}^{water}}\right)dz\times \frac{APL}{M_{lipids}m_{u}}$$

(6) $$Perm=1/\overset{z}{\underset{outside}{\int }}e^{\frac{\Delta G\left(z\right)}{RT}}dz$$

where Δ*G(z)* is the free energy at depth *z*, *R* is the molar gas constant, *T* is the thermodynamic temperature, *ρ_(z)_^water^* is the water density at depth *z* and *ρ_(n)_^water^* is the density of bulk water—the integration runs from the middle of the membrane (*z* = 0) through the membrane separated into layers parallel to the membrane plane up to the *n*th layer situated in bulk water. Thanks to the multiplying factor (where *APL* is the area per lipid, *M_lipids_* is the molecular weight of lipids, *m_u_* is the atomic mass constant) the partition coefficient log *P_lw,calc_* is in the units used in the experimental work; i.e., kg_(lipid)_/L_(water)_.

A fluid dioleoylphosphatidylcholine (DOPC) membrane model was used for lipids, as well as a skin-related solid membrane model. The DOPC membrane model is well established and the calculated partitioning into the DOPC membrane reproduces the experiment well [40]. In order to obtain structural inputs for the COSMOmic tool, the lipid bilayers were composed not only with DOPC, but also with ceramide NS24 (CER), or an equimolar mixture of CER, lignoceric acid and cholesterol (CLC). From the free energy profiles on the membranes obtained by COSMOmic, a penetration barrier (Δ*G^pen^*) and *Perm* were also extracted. Detail information about calculation models are mentioned in Appendix C.

### 3.8. Statistical Analysis

Experimental (log *P_ow,exp_*) and theoretical (log *P_ow,calc,_* log *P_lw,calc_*) hydrophobicity data were compared in order to observe correlations and differences. For the correlations, MS Excel was used to create a correlation matrix with the Analysis Toolpak add-on providing a correlation coefficient between all the calculated and measured data. Further, a linear regression was done between the data measured in the phosphate buffer at different pH and the calculated data (also in MS Excel). Apart from correlation coefficients and linear regression equations, the differences between the experimental and calculated values were evaluated as their mean differences MD.

(7) $$1/N\sum_{i}^{N}|\log P_{ow\left(lw\right),calc,i}-\log P_{ow\;,exp,i}|$$

## 4. Conclusions

In conclusion, log *P_ow_* values for selected silymarin’s flavonolignans, including their respective diastereomers and dehydro derivatives as well as flavonoids taxifolin and quercetin were experimentally determined. For the first time the data for individual diastereomers were determined. The ex vivo permeation of flavonolignans, taxifolin and quercetin through the human skin showed that none of the studied compounds reach the acceptor liquid, and all of them accumulate in the tissue. The computational prediction of log *P_ow_* values and penetration through model lipid membranes for flavonolignans, and their respective dehydro derivatives, taxifolin and quercetin were carried out and compared with experimental values. Although we observed a reliable prediction of log *P_ow_* values (r^2^ > 0.8), none of the models worked for all the experimental conditions (at various pH). Theoretical tools were unable to clearly distinguish between stereoisomers. The use of lipid membranes for the theoretical estimation of lipophilicity provides a more reliable lipophilicity prediction than log *P_ow_*. Although in silico models cannot yet predict the dermal delivery differences of compounds with very similar structural motifs (for example optical isomers) with the desired level of certainty, they can be usefully applied for estimating general lipophilicity trends and skin accumulation predictions. Therefore, the theoretically received data should be still confirmed experimentally. 

Both experimental and theoretical tools enabled the studied polyphenols to be divided into two groups based on their skin intake and lipophilicity (taxifolin, silychristin, silydianin vs. silybin, dehydrosilybin, isosilybin). This behavior should be considered during the development of dermatological preparations containing silymarin or its pure flavonolignans as biologically active components. 

## Figures and Tables

**Table 1:** Hydrophobicity (log *P_ow,exp_*) of studied compounds in phosphate buffers.

Compound	log *P_ow,exp_*				
	pH				
	4.5	5.5	6.5	7.5	8.5
Taxifolin	1.761 ± 0.017	1.763 ± 0.002	1.674 ± 0.017	1.486 ± 0.026	0.802 ± 0.046
Silychristin A	2.045 ± 0.010	2.042 ± 0.003	1.952 ± 0.017	1.786 ± 0.021	1.705 ± 0.003
Silydianin	2.076 ± 0.011	2.077 ± 0.010	1.964 ± 0.019	1.805 ± 0.017	1.742 ± 0.004
Silychristin B	2.100 ± 0.008	2.097 ± 0.003	2.003 ± 0.018	1.837 ± 0.020	1.758 ± 0.002
Dehydrosilydianin	2.178 ± 0.009	2.181 ± 0.008	2.108 ± 0.014	1.948 ± 0.024	1.843 ± 0.004
Quercetin	2.269 ± 0.010	2.266 ± 0.006	2.197 ± 0.015	1.979 ± 0.030	1.778 ± 0.005
Silybin A	2.314 ± 0.008	2.310 ± 0.010	2.180 ± 0.024	1.972 ± 0.025	1.872 ± 0.002
Silybin B	2.346 ± 0.006	2.336 ± 0.009	2.203 ± 0.019	1.999 ± 0.023	1.904 ± 0.002
Dehydrosilychristin	2.425 ± 0.003	2.420 ± 0.007	2.352 ± 0.016	2.162 ± 0.029	2.025 ± 0.010
Isosilybin A	2.432 ± 0.002	2.422 ± 0.008	2.289 ± 0.024	2.081 ± 0.023	1.992 ± 0.002
Isosilybin B	2.458 ± 0.005	2.444 ± 0.010	2.306 ± 0.026	2.100 ± 0.022	2.014 ± 0.002
Dehydrosilybin	2.948 ± 0.003	2.932 ± 0.019	2.788 ± 0.036	2.508 ± 0.038	2.353 ± 0.019
Dehydroisosilybin	3.154 ± 0.002	3.131 ± 0.003	2.931 ± 0.047	2.619 ± 0.039	2.462 ± 0.026

The letters A or B in the name of compounds mean that diastereomers of the compound exist and they were separated by a chromatographic system under the applied experimental conditions. Data are presented as mean ± S.D., *n* = 4.

**Table 2:** Hydrophobicity of studied compounds predicted by computational tools as octanol/water partition coefficient (log *P_ow,calc_*) and lipid/water partition coefficient (log *P_lw,calc_*) in membrane models.

Compound	log *P_ow,calc_*				log *P_lw,calc_*		
	COSMOtherm	Molinspiration	AlogP		CER	DOPC	CLC
Taxifolin	1.01	0.71	1.07		1.14	1.49	0.22
Silychristin A	2.55	1.26	2.13		1.64	2.35	1.10
Silydianin	1.29	1.21	1.84		2.28	2.25	1.51
Silychristin B	2.51	1.26	2.13		1.76	2.35	1.06
Dehydrosilydianin	1.07	1.21	1.84		2.13	2.08	1.34
Quercetin	2.17	1.68	1.81		1.80	2.17	0.79
Silybin A	3.30	1.47	2.35		2.21	3.06	1.93
Silybin B	3.20	1.47	2.35		2.19	2.90	1.83
Dehydrosilychristin	3.61	2.24	2.57		2.26	3.09	1.50
Isosilybin A	3.56	1.47	2.35		2.82	3.44	2.47
Isosilybin B	3.25	1.47	2.35		2.46	3.17	2.16
Dehydrosilybin	4.17	2.44	2.80		2.78	3.68	2.57
Dehydroisosilybin	4.18	2.44	2.81		3.42	3.97	3.09

CER—ceramide, DOPC—dioleoylphosphatidylcholine, CLC—ceramide, lignoceric acid and cholesterol mixture. The letters A or B in the name of a compound mean that diastereomers of the compound exists and they were calculated separately in the computational tools.

**Table 3:** Skin intake of studied compounds evaluated by transdermal transport.

Compound		Concentration (pmol g^−1^ of Skin)							
		pH 6.5				pH 8.5			
	EtOH (%, *v*/*v*)					EtOH (%, *v*/*v*)			
	0	5	10	15		0	5	10	15
Taxifolin	3101	4237	2068	2228		2099	1518	1202	2022
Silychristin	16717	8655	5431	5690		5358	5166	2912	3275
Silydianin	6997	6606	7809	3457		4966	2204	6257	6129
Silybin	53093	39744	15984	31531		17676	26824	13258	7311
Quercetin	56230	78622	28628	49162		6586	6185	10935	2699
Isosilybin	77643	71638	47375	29113		31985	20131	8591	7969
Dehydrosilybin	45105	45145	22399	35810		50248	30352	28477	26721

The studied compounds were applied to the skin membrane at a concentration of 50 µM in the donor liquid and incubated at 32 °C. After 24 h, the skin was collected for evaluating the content of the studied compounds.

**Table 4:** Permeability (log *Perm*) and penetration barrier (Δ*G^pen^*) calculated from COSMOmic free energy profiles in membrane models.

Compound	log *Perm*				Δ*G^pen^* (kcal/mol)		
	CER	DOPC	CLC		CER	DOPC	CLC
Taxifolin	−4.56	−1.44	−4.67		9.39	5.30	7.78
Silychristin	−2.41	−0.35	−2.53		6.96	4.69	5.96
Silydianin	−3.40	−0.95	−3.31		9.29	6.02	7.98
Silybin A	−1.01	−0.28	−1.07		5.49	3.59	5.08
Silybin B	−1.05	−0.30	−1.13		5.77	3.45	5.07
Quercetin	−3.11	−0.25	−3.23		8.24	4.37	6.41
Isosilybin A	−0.51	−0.29	−0.56		5.97	2.88	5.10
Dehydrosilybin	−0.28	−0.26	−0.24		4.55	2.88	4.07

CER—ceramide, DOPC—dioleoylphosphatidylcholine, CLC—ceramide, lignoceric acid and cholesterol mixture. The letters A or B for silybin mean that calculations were done for its respective diastereomers.

## Appendix A

**Table A1:** Reference compounds’ log *P_ow_* values and composition of reference compound mixture.

Reference Compound	log *P_ow_*	Amount in Mixture
Aniline	0.9	2 µl L^−1^
Phenol	1.5	10 ng L^−1^
4-Chloroaniline	1.8	10 ng L^−1^
Nitrobenzene	1.9	10 ng L^−1^
Benzene	2.1	30 µl L^−1^
Trichloroethylene	2.4	50 µl L^−1^
Toluene	2.7	30 µl L^−1^
Chlorobenzene	2.8	50 µl L^−1^
Naphthalene	3.6	5 ng L^−1^

**Table A2:** Relation between capacity factor (*k*) and log *P_ow_* of reference compounds in phosphate buffer.

pH	Linear Equitation	r^2^	Quadratic Equitation	r^2^
4.5	y = 15.564x − 9.2141	0.8970	y = −7.6607x^2^ + 54.46x − 55.269	0.9953
5.5	y = 14.935x − 6.5672	0.8942	y = −7.5071x^2^ + 53.051x − 51.698	0.9964
6.5	y = 14.855x − 6.4229	0.8965	y = −7.3626x^2^ + 52.238x − 50.685	0.9962
7.5	y = 14.895x − 6.2264	0.8924	y = −7.5201x^2^ + 53.077x − 51.435	0.9954
8.5	y = 14.99x − 6.7367	0.8925	y = −7.5849x^2^ + 53.501x − 52.335	0.9959

The separation of reference compounds was carried out in 5 mM phosphate buffer with MeOH (5%, *v*/*v*), pH was set in the range of 4.5–8.5 (HPLC mobile phase A) in a linear gradient with MeOH (100%; HPLC mobile phase B) at 25 °C. For further conditions, see Materials and Methods section.

**Table A3:** Hydrophobicity (log *P_ow,exp_*) of studied compounds in formate buffers.

Compound	log *P_ow,exp_*				
	pH				
	4.5	5.5	6.5	7.5	8.5
Taxifolin	1.776 ± 0.000	1.763 ± 0.000	1.704 ± 0.000	1.572 ± 0.002	0.714 ± 0.007
Silychristin A	2.059 ± 0.007	2.040 ± 0.000	2.003 ± 0.001	1.925 ± 0.002	1.714 ± 0.001
Silydianin	2.088 ± 0.003	2.071 ± 0.000	2.025 ± 0.000	1.935 ± 0.001	1.734 ± 0.000
Silychristin B	2.114 ± 0.007	2.096 ± 0.000	2.060 ± 0.000	1.986 ± 0.002	1.778 ± 0.006
Dehydrosilydianin	2.187 ± 0.001	2.175 ± 0.000	2.154 ± 0.000	2.104 ± 0.000	1.872 ± 0.001
Quercetin	2.279 ± 0.001	2.266 ± 0.000	2.248 ± 0.001	2.203 ± 0.001	1.895 ± 0.000
Silybin A	2.318 ± 0.001	2.302 ± 0.000	2.272 ± 0.000	2.200 ± 0.001	1.920 ± 0.000
Silybin B	2.350 ± 0.001	2.335 ± 0.000	2.304 ± 0.001	2.229 ± 0.003	1.959 ± 0.000
Dehydrosilychristin	2.433 ± 0.002	2.421 ± 0.000	2.404 ± 0.005	2.383 ± 0.000	2.183 ± 0.001
Isosilybin A	2.437 ± 0.001	2.422 ± 0.000	2.397 ± 0.000	2.328 ± 0.000	2.059 ± 0.000
Isosilybin B	2.459 ± 0.001	2.444 ± 0.000	2.415 ± 0.000	2.348 ± 0.001	2.081 ± 0.001
Dehydrosilybin	2.947 ± 0.003	2.936 ± 0.001	2.928 ± 0.001	2.898 ± 0.007	2.661 ± 0.010
Dehydroisosilybin	3.161 ± 0.009	3.143 ± 0.002	3.134 ± 0.004	3.096 ± 0.004	2.815 ± 0.004

The letters A or B in the name of a compound mean that diastereomers of the compound exist and they were separated by a chromatographic system under the applied experimental conditions. Data are presented as mean ± S.D., *n* = 4.

**Table A4:** Hydrophobicity (log *P_ow,exp_*) of studied compounds in acetate buffers.

Compound	log *P_ow,exp_*				
	pH				
	4.5	5.5	6.5	7.5	8.5
Taxifolin	1.774 ± 0.000	1.765 ± 0.001	1.710 ± 0.001	1.577 ± 0.006	0.783 ± 0.013
Silychristin A	2.051 ± 0.001	2.042 ± 0.000	1.999 ± 0.001	1.920 ± 0.007	1.733 ± 0.000
Silydianin	2.083 ± 0.001	2.075 ± 0.005	2.017 ± 0.000	1.928 ± 0.008	1.757 ± 0.000
Silychristin B	2.106 ± 0.001	2.097 ± 0.001	2.054 ± 0.000	1.980 ± 0.007	1.793 ± 0.000
Dehydrosilydianin	2.183 ± 0.001	2.178 ± 0.002	2.151 ± 0.001	2.089 ± 0.008	1.894 ± 0.001
Quercetin	2.273 ± 0.001	2.268 ± 0.000	2.245 ± 0.001	2.202 ± 0.009	1.919 ± 0.003
Silybin A	2.315 ± 0.000	2.307 ± 0.006	2.257 ± 0.001	2.185 ± 0.008	1.942 ± 0.001
Silybin B	2.347 ± 0.001	2.337 ± 0.001	2.289 ± 0.001	2.217 ± 0.006	1.979 ± 0.000
Dehydrosilychristin	2.429 ± 0.002	2.424 ± 0.001	2.405 ± 0.004	2.390 ± 0.004	2.203 ± 0.003
Isosilybin A	2.434 ± 0.001	2.428 ± 0.005	2.377 ± 0.001	2.310 ± 0.010	2.079 ± 0.000
Isosilybin B	2.455 ± 0.001	2.446 ± 0.000	2.396 ± 0.001	2.330 ± 0.010	2.102 ± 0.000
Dehydrosilybin	2.943 ± 0.001	2.940 ± 0.004	2.909 ± 0.003	2.888 ± 0.015	2.677 ± 0.000
Dehydroisosilybin	3.153 ± 0.002	3.148 ± 0.007	3.110 ± 0.002	3.074 ± 0.021	2.840 ± 0.005

The letters A or B in the name of a compound mean that diastereomers of the compound exist and they were separated by a chromatographic system under the applied experimental conditions. Data are presented as mean ± S.D., *n* = 4.

## Appendix B

**Table A5:** Correlation coefficient (r^2^) and mean differences (MD) of *n*-octanol/water partition coefficient (log *P_ow,calc_*) to log *P_ow,exp_* measured at different pH and calculated lipid/water partition coefficient (log *P_lw,calc_*) in model membranes.

Parameter	pH	log *P_ow,calc_*				log *P_lw,calc_*		
		COSMOtherm	Molinspiration	AlogP		CER	DOPC	CLC
r^2^	4.5	0.68	0.83	0.74		0.82	0.84	0.79
	5.5	0.68	0.83	0.74		0.82	0.84	0.79
	6.5	0.67	0.87	0.74		0.79	0.81	0.75
	7.5	0.66	0.87	0.77		0.81	0.82	0.76
	8.5	0.61	0.71	0.87		0.78	0.77	0.75
MD	4.5	0.83	0.78	0.21		0.26	0.49	0.69
	5.5	0.84	0.78	0.21		0.26	0.50	0.69
	6.5	0.90	0.66	0.19		0.24	0.58	0.62
	7.5	1.02	0.47	0.27		0.32	0.75	0.51
	8.5	1.08	0.35	0.32		0.37	0.90	0.44

**Table A6:** Correlation coefficients (r^2^) between experimental *n*-octanol/water partition coefficients (log *P_ow,exp_*) and skin intake at pH 6.5 and 8.5.

		Log of Skin Intake, pH 6.5 (without DSB)				Log of Skin Intake, pH 8.5			
		% of Ethanol in Donor Liquid				% of Ethanol in Donor Liquid			
	**pH**	**0**	**5**	**10**	**15**	**0**	**5**	**10**	**15**
Log *P_ow,exp_*	6.5	0.54 (0.94)	0.55 (0.88)	0.57 (0.91)	0.60 (0.85)	-	-	-	-
	8.5	-	-	-	-	0.77	0.63	0.83	0.67

- Only results obtained at the same pH were included in the correlation.

**Table A7:** Correlation coefficients (r^2^) between skin intake and calculated permeability (log *Perm*), lipid/water partition coefficient (log *P_lw,calc_*) and penetration barrier (Δ*G^pen^*) in membrane models.

		Skin Intake, log Units, pH 6.5 (without QU)				Skin Intake, log Units, pH 8.5			
		% of Ethanol in Donor Liquid				% of Ethanol in Donor Liquid			
		0	5	10	15	0	5	10	15
log Perm	CER	0.95	0.93	0.82	0.94	0.94	0.94	0.76	0.63
	DOPC	0.87	0.69	0.61	0.75	0.68	0.79	0.57	0.40
	CLC	0.94	0.92	0.83	0.94	0.95	0.93	0.79	0.66
log *P_lw,calc_*	CER	0.61	0.66	0.87	0.58	0.80	0.52	0.74	0.75
	DOPC	0.84	0.87	0.85	0.86	0.97	0.83	0.81	0.76
	CLC	0.76	0.81	0.90	0.76	0.92	0.71	0.84	0.79
Δ*G^pen^* (kcal/mol)	CER	0.84	0.80	0.56	0.89	0.82	0.94	0.65	0.53
	DOPC	0.82	0.89	0.64	0.88	0.82	0.87	0.50	0.44
	CLC	0.83	0.79	0.56	0.88	0.82	0.92	0.61	0.52

CER—ceramide, DOPC—dioleoylphosphatidylcholine, CLC—ceramide, lignoceric acid and cholesterol mixture. The cells are coloured according to the r^2^ values based on the displayed colour scale. The linear regression equations for skin intake at pH 8.5 on model membranes were: CER: log (Intake to skin) = 0.76 * log *P_lw,calc,CER_* + 2.43. DOPC: log (Intake to skin) = 0.63 * log *P_lw,calc,DOPC_* + 2.36. CLC: log (Intake to skin) = 0.54 * log *P_lw,calc,CLC_* + 3.20.

## Appendix C

### Calculation Details:

In order to obtain a computational prediction of the hydrophobicity of flavonolignans, TA, and QU, the *n*-octanol-water partition coefficients were calculated (log *P_ow,calc_*) with the commercial software COSMOtherm 15 software [18] based on quantum-chemical calculations and by the freely available online tools AlogP [15] and Molinspiration [16].

First, the molecular structures were downloaded as pdf files from Pubchem [41]. For COSMOtherm calculations, the same workflow was used as recently in Navrátilová et al. [42]. Individual conformers were generated using the Schrodinger software package [43,44] and for each conformer its vacuum energy and sigma-surfaces were calculated. These were calculated at the BP/TZVP-fine level of theory both for vacuum and cosmo solvent calculation with Turbomole 6.3 [45]. For the Log *P_ow_* calculation, COSMOtherm provides a pre-defined setup for partitioning between two fluid phases, where octanol is modelled as a wet octanol, meaning 27.4% of its mass is water, reflecting experimentally more relevant conditions than using dry 100% octanol. The partitioning calculation was performed at 25 and 32 °C. Generally, COSMOtherm calculates the thermodynamic properties of fluid phases, including the partition of substances between variously composed phases. However, a full description of COSMOtherm’s principles is outside the scope of this paper and was described; e.g., by Klamt [46].

For lipid/water partition coefficient calculations, we used COSMOmic [39]. The semi-continuous tool of COSMOtherm uses a lipid bilayer structure/s as an input, separates them into layers and calculates the partition coefficient of the drug into each location (into each layer taking into account the possibility of interactions of the drug with neighboring layers by its rotation). The partition coefficients into individual locations can be easily converted into a free energy profile or into the general lipid/water partition coefficient log *P_lw_*. Fluid dioleoylphosphatidylcholine (DOPC) membrane models as well as skin-related solid membrane models were used. The DOPC membrane model is well established and the calculated overall partitioning into phosphatidylcholine membranes reproduces the experiment well. The solid membrane models, ceramide-based ones, do not reflect the basic COSMOtherm requirement for work with fluid phases, but can be also used for estimation drug/lipid interactions, taking into account the need for free energy barrier re-evaluation.

Our previous MD simulations of lipid bilayers consisted of DOPC, ceramide NS24 (CER) and an equimolar mixture of ceramide NS24, lignoceric acid and cholesterol (referred to below as the CLC-mixture) were used. The distribution of individual lipid and water atoms during the last 100 ns of simulations and the averaged atomic distributions of each of the membranes were monitored. Further, cosmo surfaces (the inducted charge distribution of surface segments) on each of the lipid types (BP/TZVP-fine level of theory) were calculated and COSMOmic was run. The permeability coefficient log *Perm* was also extracted from the free energy profiles on the membranes obtained with COSMOmic.
